# *SIGMAR1* Knockdown Enhances Oral Cancer Cell Chemosensitivity to Cisplatin via Decreased *PD-L1* Expression

**DOI:** 10.3390/ijms252211856

**Published:** 2024-11-05

**Authors:** Pablo Shimaoka Chagas, Cristiana Bernadelli Garcia, Lucas Oliveira Sousa, Gabriel da Silva, Graziella Ribeiro de Sousa, Rodolfo Cabral Marcelino, Leandro Luongo de Matos, Luiz Paulo Kowalski, Évila Salles, Lei Wang, Babak Baban, Andréia Machado Leopoldino

**Affiliations:** 1 Department of Clinical Analyses, Toxicology and Food Sciences, School of Pharmaceutical Sciences of Ribeirão Preto, University of São Paulo, Av. do Café s/n, Ribeirão Preto 14040-903, SP, Brazil; crisdelli@fcfrp.usp.br (C.B.G.); losousa@usp.br (L.O.S.); biel-189@hotmail.com (G.d.S.); graziellasousa@usp.br (G.R.d.S.); andreiaml@usp.br (A.M.L.); 2 Department of Oral Biology and Diagnostic Services, Dental College of Georgia, Augusta University, Augusta, GA 30912, USA; esalles@augusta.edu (É.S.); lewang@augusta.edu (L.W.); bbaban@augusta.edu (B.B.); 3 Laboratory of Molecular Modeling and Computer Simulation/MolMod-CS, Institute of Chemistry, Federal University of Alfenas, Alfenas 37130-001, MG, Brazil; rodolfo_cabralcm@hotmail.com; 4 Head and Neck Surgery Department, Instituto do Câncer do Estado de São Paulo, Hospital das Clínicas, University of São Paulo Medical School (ICESP HCFMUSP), São Paulo 01246-000, SP, Brazil; l.matos@fm.usp.br; 5 Faculdade Israelita de Ciências da Saúde Albert Einstein, São Paulo 05653-120, SP, Brazil; 6 Head and Neck Surgery Department, University of São Paulo Medical School, São Paulo 05403-000, SP, Brazil; lp_kowalski@uol.com.br; 7 Head and Neck Surgery Department, A. C. Camargo Cancer Center, São Paulo 01525-001, SP, Brazil; 8 Department of Neurology, Medical College of Georgia, Augusta University, Augusta, GA 30912, USA

**Keywords:** *SIGMAR1*, *PD-L1*, oral cancer, immunotherapy

## Abstract

Emerging evidence suggests that aberrant expression levels of Sigma1 (*SIGMAR1*, also known as sigma-1 receptor) have been implicated in the progression of various diseases, including cancer. However, its significance in oral cancer (OC) has not been thoroughly explored. To advance in this field, the present study aimed to investigate the impact of *SIGMAR1* knockdown in oral cancer cells. To do so, we included in this study our cohort of human OC samples and OC cell lines, which were utilized for experimental verification through several in vitro assays. Our findings revealed that *SIGMAR1* overexpression was associated with poor survival rates and positively correlated with *PD-L1* overexpression in human oral cancer samples. Furthermore, *SIGMAR1* inhibition led to a decrease in *PD-L1* expression and sensitized oral cancer cells to cisplatin treatment by enhancing apoptosis. These results suggest that *SIGMAR1* knockdown may present a promising strategy worthy of further exploration in the management of oral cancer.

## 1. Introduction

Oral squamous cell carcinoma (OSCC), commonly referred to as oral cancer (OC), can be a life-threatening disease and is one of the major types of head and neck squamous cell carcinoma (HNSCC) [1]. The diagnosis usually occurs over the age of 50 years, and the standard treatment is surgery, radiotherapy, chemotherapy, and/or immunotherapy [1]. Unfortunately, the reported 5-year overall survival (OS) rate is lower than 60% [2]. Consequently, the main reason for treatment failure is locoregional recurrence, which is possibly associated with our limited molecular understanding of OC tumorigenesis. Furthermore, the surviving patients often suffer from severe treatment-related side effects [3]. Recently, new insights into the influence of the tumor immune response on OC aggressiveness have been reported [4]. Nevertheless, the main targets of these cross-talks between cancer cells and tumor immune microenvironments remain undiscovered.

To address this knowledge gap, we highlighted the Sigma-1 receptor (SIGMAR1), encoded by the *SIGMAR1* gene (9p13.3). Notably, SIGMAR1 plays multifaceted roles in both physiological and pathological conditions [5]. Under normal physiological conditions, SIGMAR1 is involved in cellular processes, such as the modulation of ion channels, regulation of calcium signaling, and maintenance of cellular homeostasis. It is known for its neuroprotective functions, including the enhancement of neuroplasticity, attenuation of neuroinflammation, and protection against oxidative stress [6]. Pathologically, the dysregulation of SIGMAR1 has been implicated in various neurodegenerative diseases, such as Alzheimer’s and Parkinson’s disease, where its malfunction can exacerbate neuronal damage and cognitive deficits. On the other hand, it was also reported that SIGMAR1 protein also plays an intriguing role in the immunomodulatory process (immunity and inflammation) and cancer progression [5,6].

In the context of tumorigenesis and cancer progression, SIGMAR1 has garnered attention due to its potential involvement in cancer biology. Recent studies have highlighted its functions in different types of cancers, indicating that SIGMAR1 might influence cancer cell survival, proliferation, and metastasis [5]. For instance, in breast cancer, SIGMAR1 has been shown to promote cell proliferation and survival, while in prostate cancer, its expression correlates with increased tumor growth and resistance to apoptosis [5]. Additionally, evidence suggests that SIGMAR1 may modulate the tumor microenvironment, influencing factors such as angiogenesis and immune evasion [5,6].

Recently, it was also demonstrated that *SIGMAR1* modulates the programmed death-ligand 1 (*PD-L1*) expression by autophagy [7]. Notably, *PD-L1* is a critical immune checkpoint protein that binds to programmed death 1 (PD-1) on T cells, leading to tumor immune escape and, consequently, increased immunosuppression, cancer aggressiveness, and drug resistance [8]. Curiously, in the last decade the potential function of *SIGMAR1* in cancer has gained increasing interest as a potential new biomarker, as previously demonstrated in breast cancer [9], colorectal cancer [10], and prostate cancer [11]. Nevertheless, the impact of SIGMAR1 on oral cancer is largely unknown.

In our study, we observed that *SIGMAR1* overexpression was linked to unfavorable survival rates and was positively correlated with *PD-L1* overexpression in human oral cancer samples. Importantly, the inhibition of *SIGMAR1* led to a reduction in *PD-L1* abundance at both the total protein and surface expression levels. Consequently, this inhibition decreased chemoresistance to cisplatin and promoted apoptosis in human OC cells. Overall, our study provides valuable insights into the role of *SIGMAR1* in oral cancer pathogenesis and suggests that *SIGMAR1*-targeted interventions could represent a promising avenue for future clinical trials and therapeutic development in the field of oral cancer treatment.

## 2. Results

### 2.1. SIGMAR1 Up-Regulated Is Correlated with PD-L1 Overexpression in Oral Cancer

**Figure 1:**
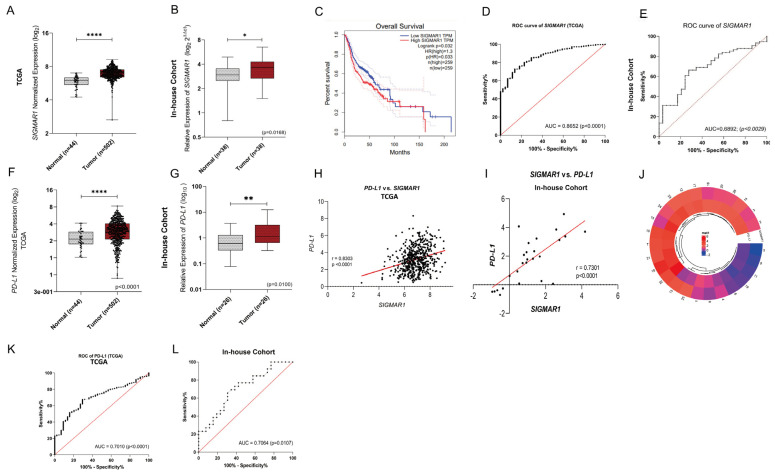
*SIGMAR1* is overexpressed and positively correlated with *PD-L1* expression in human oral cancer. (**A**) *SIGMAR1* levels were compared between normal tissues (*n* = 44) and OC samples (*n* = 502) using TCGA data and Mann–Whitney test (**** *p* = 0.001). (**B**) Comparison of *SIGMAR1* levels between OC (*n* = 26) and paired non-tumor tissues (*n* = 26) in a Brazilian cohort. Mann–Whitney test (* *p* < 0.0168). (**C**) Kaplan–Meier survival curves show a worse prognosis associated with high levels of *SIGMAR1* in patients with oral cancer. (**D**) ROC curve analysis of *SIGMAR1* in OC using TCGA data. (**E**) ROC curve analysis of *SIGMAR1* in OC using in-house cohort data. (**F**) *PD-L1* levels were compared between normal tissues (*n* = 44) and OC samples (*n* = 502) using TCGA data and Mann–Whitney test (**** *p* < 0.0001). (**G**) Comparison of *PD-L1* levels between OC (*n* = 26) and paired non-tumor tissues (*n* = 26) in a Brazilian cohort. Mann–Whitney test (** *p* < 0.01). (**H**–**J**) Gene expression correlation analyses. Spearman’s correlation analysis showing gene expression levels of *SIGMAR1* and *PD-L1* in OC patients from TCGA data and from a Brazilian cohort. (**K**) ROC curve analysis shows the predictive value of *PD-L1* in OC: TCGA data. *p* < 0.0001. (**L**) ROC curve analysis shows the predictive value of *PD-L1* in OC: Brazilian cohort. *p* = 0.0107.

To examine the SIGMAR1 expression pattern in oral cancer, we used bulk RNA sequencing from TCGA data and our in-house cohort, which included 26 primary cases of OC samples. As a result, the overexpression of SIGMAR1 was observed in tumors vs. adjacent non-tumor tissues in the TCGA data (*p* < 0.001) and confirmed in our in-house cohort study (*p* < 0.016) (Figure 1A,B). Thus, survival data analysis was performed, and it revealed that high levels of SIGMAR1 (mRNA) are associated with a significantly worse prognosis for oral cancer patients (Figure 1C, *p* = 0.033). Furthermore, ROC analysis was employed to provide the reliable diagnostic efficiency of SIGMAR1 as a potential biomarker for OC using both TCGA and our in-house cohort data. As shown in Figure 1D,E, the areas under the ROC curve were 0.8652 (*p* < 0.0001) and 0.6892 (*p* = 0.0029), respectively, suggesting a significantly high precision of SIGMAR1 in discriminating tumors from non-tumor tissues. Nonetheless, these findings suggest that the SIGMAR1 gene may be considered a new potential biomarker for OC.

Next, we also evaluated the PD-L1 gene expression using the TCGA data and our in-house cohort. As a result, higher PD-L1 mRNA levels were observed in the OC samples than in the paired non-tumor tissues, in both the TCGA data (Figure 1F, *p* < 0.0001) and our in-house cohort (Figure 1G, *p* = 0.0100) by quantitative qRT-PCR. Our results also demonstrated that increased SIGMAR1 mRNA expression was positively correlated with the high expression levels of PD-L1, by using the TCGA data (Figure 1H, rho = 0.8303, *p* < 0.0001) and our in-house cohort data (Figure 1I, rho = 0.7301, *p* < 0.0001). We then detailed the SIGMAR1 and PD-L1 expression analysis using a heatmap correlation plot. For instance, according to the heatmap, we could observe that in most human oral cancer samples, SIGMAR1 had a correlation profile with the PD-L1 expression values (Figure 1J), in our cohort. Furthermore, ROC analysis was employed to provide the reliable diagnostic efficiency of PD-L1 as a potential biomarker for oral cancer. As shown in Figure 1K, the area under the ROC curve was 0.7010 (*p* < 0.0001), which also suggests a significantly high precision of PD-L1 in discriminating OC tissues from non-tumor tissues by using TCGA data. To our surprise, the same analysis using our in-house cohort data also demonstrated that the area under the ROC curve was 0.7064 (*p* = 0107), corroborating the results observed using TCGA data, as shown Figure 1L. Taken together, we provide evidence here that SIGMAR1 can be a positive regulator of PD-L1 overexpression in OC samples.

### 2.2. The Knockdown of SIGMAR1 Decreased PD-L1 Expression in Human Oral Cancer Cells

**Figure 2:**
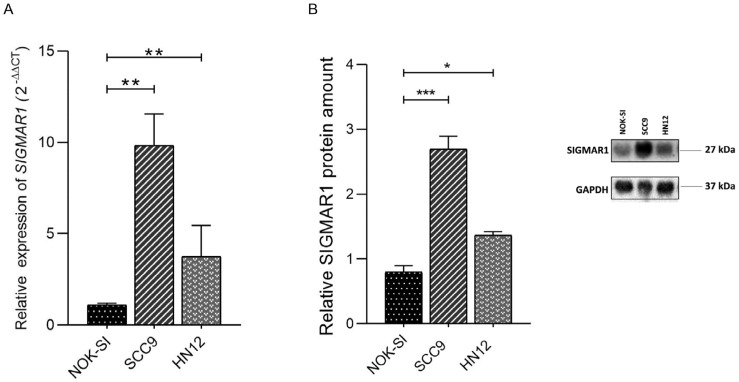
Evaluation of SIGMAR1 expression profile in oral cancer cells. (**A**) Relative expression of mRNA *SIGMAR1* levels in NOK-SI, SCC9, and HN12 cell lines by RT-qPCR. The graph shows the mean ± standard deviation of three independent experiments (** *p* < 0.01). (**B**) Western blot and protein relative quantification of SIGMAR1 in NOK-SI, SCC9, and HN12 cell lines. Relative protein quantification was performed using ImageJ software, version 1.54. GAPDH protein was used as an endogenous control. Data are shown as mean ± standard deviation (SD) of three independent experiments. * *p* < 0.05 and *** *p* < 0.001.

Firstly, we evaluated the SIGMAR1 expression profiles in non-tumor oral keratinocytes spontaneously immortalized (NOK-SI) and in two human OC lines, SCC9 and HN12, by quantitative PCR and Western blot. As result, the SCC9 (*p* < 0.001) and HN12 (*p* < 0.001) cells showed an increased expression of SIGMAR1 at the mRNA and protein levels when compared to NOK-SI (Figure 2A,B).

**Figure 3:**
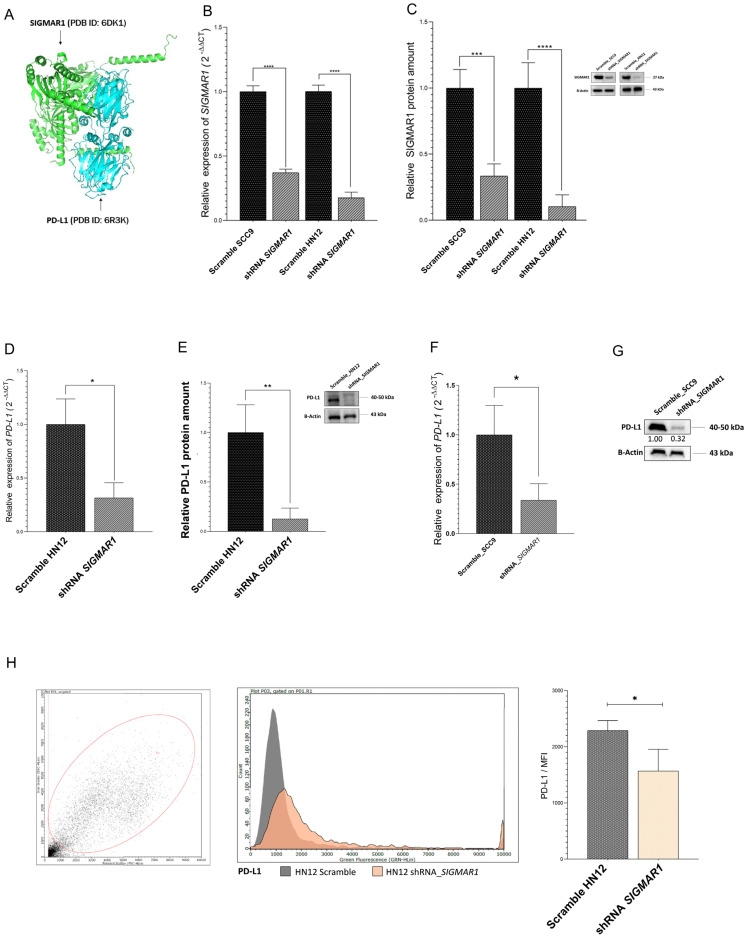
*SIGMAR1* knockdown decreased *PD-L1* expression in oral cancer cells. (**A**) Three-dimensional representation of molecular docking analysis between the SIGMAR1 and PD-L1. The interacting residues are displayed as green (SIGMAR1) and blue (PD-L1). (**B**) *SIGMAR1* silencing in shRNA SCC9 and shRNA HN12 cell lines, respectively, compared to control (shRNA Scramble) by RT-qPCR. The graph shows the mean ± standard deviation of three independent experiments (**** *p* < 0.0001). (**C**) Western blot and protein relative quantification showing reduced levels of SIGMAR1 in shRNA SCC9 and shRNA HN12 cell lines compared to control (shRNA Scramble). Relative protein quantification was performed using ImageJ software. β-actin protein was used as an endogenous control. Data are shown as mean ± standard deviation (SD) of three independent experiments. * *p* < 0.05, compared to control (shRNA Scramble) (*** *p* <0.001 and **** *p* < 0.0001). (**D**) *PD-L1* mRNA expression in *SIGMAR1* shRNA HN12 cell lines compared to control (shRNA Scramble) by RT-qPCR. The graph shows the mean ± standard deviation of three independent experiments (* *p* < 0.05). (**E**) Western blot and protein relative quantification showing reduced levels of PD-L1 in SIGMAR1 shRNA HN12 cell lines compared to control (shRNA Scramble). Relative protein quantification was performed using ImageJ software. β-actin protein was used as an endogenous control. Data are shown as mean ± standard deviation (SD) of three independent experiments. ** *p* < 0.01, compared to control (shRNA Scramble). (**F**) *PD-L1* mRNA expression in *SIGMAR1* shRNA SCC9 cell lines compared to control (shRNA Scramble) by RT-qPCR. The graph shows the mean ± standard deviation of three independent experiments (* *p* < 0.05). (**G**) Western blot and protein relative quantification showing reduced levels of PD-L1 in SIGMAR1 shRNA SCC9 cell lines compared to control (shRNA Scramble). Relative protein quantification was performed using ImageJ software. β-actin protein was used as an endogenous control. (**H**) The gating strategies of flow cytometric analysis and the analysis of PD-L1 expression on the *SIGMAR1* shRNA HN12 cell lines compared to control (shRNA Scramble) cells and the statistical summary for PD-L1 expression on the *SIGMAR1* shRNA HN12 cell lines compared to control (shRNA Scramble) cells. Data are shown as mean ± standard deviation (SD) of three independent experiments. * *p* < 0.05.

Initially, we found that SIGMAR1 expression was positively correlated with PD-L1 expression. Afterwards, to investigate whether SIGMAR1 was a direct PD-L1 target, the ClusPro 2.0 server (https://cluspro.org) was used to determine their binding affinity and the protein–protein interaction of SIGMAR1 (PDB ID:6DK1) and the PD-L1 (PDB ID:6R3K). The three-dimensional structure of the SIGMAR1 receptor ligand binding region with PD-L1 was visualized by the PyMOL molecular graphics system, as shown in Figure 3A. The results showed that SIGMAR1 had an excellent binding affinity and that it interacted with PD-L1 (CW: −1355.6, Kcal/mol). Based on this, the next step was performed: the experimental validation of these results in human oral cancer cell lines.

Thus, we selected these two different oral cancer cell lines with small hairpin RNAs (shRNAs) targeting SIGMAR1. Additionally, an empty vector (shRNA Scramble) was obtained as a negative control. In particular, the qRT-PCR and Western blot analysis showed that the silencing effect of SIGMAR1 was more pronounced in the HN12 cells when compared to the SCC9 cells and was approximately 80% and 90% at the mRNA and protein levels, respectively, relative to the control (Figure 3B,C, *p* < 0.0001).

Next, we aimed to evaluate the PD-L1 mRNA and protein levels in the HN12 and SCC9 SIGMAR1 knockdown cells, respectively. As a result, the qRT-PCR (*p* < 0.01) and Western blot analysis (*p* < 0.001) showed that the silencing of SIGMAR1 decreased the PD-L1 expression, by approximately 70% and 90% at the mRNA and protein levels, respectively, relative to the control in the HN12 cells (Figure 3D,E, *p* < 0.001). Consistently, the knockdown of SIGMAR1 significantly reduced the PD-L1 expression, by approximately 65% and 68% at the mRNA and protein levels, respectively, relative to the control in the SCC9 cells, as shown in Figure 3F,G, *p* < 0.001.

As noted, these results were also more pronounced in the HN12 cells when compared to the SCC9 cells. Thus, the HN12 cells shRNA_SIGMAR1 and the control group were used, and by flow cytometry, we also found that SIGMAR1 knockdown significantly decreased PD-L1 protein exposition at the surface of human oral cancer cell line compared to the control group (Figure 3H, *p* < 0.001). All these findings suggest that SIGMAR1 may promote immune escape, possibly by upregulating PD-L1 expression in oral cancer cells.

### 2.3. SIGMAR1 Knockdown Sensitizes Chemoresistance to Cisplatin and Increases Cell Death by Apoptosis

**Figure 4:**
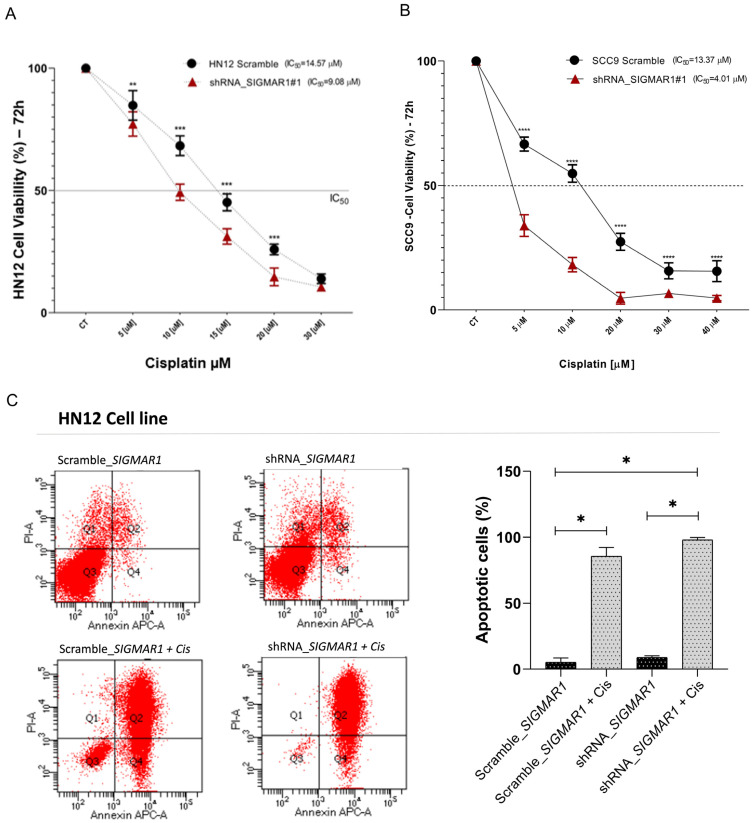
*SIGMAR1* knockdown promotes cisplatin chemosensitivity while enhancing apoptosis in human oral cancer cells. (**A**) Cell viability assay showing a reduction in *SIGMAR1* shRNA HN12 cell line cell viability after cisplatin treatment (5, 10, 15, 20, and 30 µM) for 72 h compared to control (shRNA Scramble). The IC50 values of cisplatin treatment in *SIGMAR1* shRNA HN12 cell line and HN12 Scramble cell line are 9.08 µM and 14.57 µM, respectively (** *p* < 0.01 and *** *p* < 0.001). (**B**) Cell viability assay in SCC9 cell line. The results show a reduction in SIGMAR1 shRNA SCC9 cell line cell viability after cisplatin treatment (5, 10, 15, 20, 30, and 40 µM) for 72 h compared to control (shRNA Scramble). The IC50 values of cisplatin treatment in SIGMAR1 shRNA SCC9 cell line and SCC9 Scramble cell line are 4.01 µM and 13.37 µM, respectively (**** *p* < 0.0001). (**C**) *SIGMAR1* knockdown associated with cisplatin treatment increased apoptosis in *SIGMAR1* shRNA HN12 cell line compared to control (shRNA Scramble). Apoptosis was detected by flow cytometry after Annexin and propidium iodide staining. Two-way ANOVA test. Data are shown as mean ± standard deviation (SD) of three independent experiments. * *p* < 0.05.

We also evaluated whether SIGMAR1 expression is involved in viability and chemoresistance by using the HN12 cells. As a result, the HN12 SIGMAR1 knockdown cells were more sensitive to chemotherapy compared to the Scramble cells (*p* < 0.001) when treated with different concentrations (5, 10, 20, 30, and 40 µM) of cisplatin for 72 h. The SIGMAR1 knockdown cells exhibited a 50% inhibitory effect (IC50) with a concentration of 9.08 µM compared to an IC50 with 14.57 µM of the control Scramble in the HN12 cells (Figure 4A). Additionally, we conducted the same analyses for the SCC9 lineage to reinforce the initial evidence observed in the HN12 cells. As shown in Figure 4B, the SIGMAR1 knockdown cells were more sensitive to chemotherapy and exhibited a 50% inhibitory effect (IC50) at a concentration of 4.01 µM, compared to an IC50 of 13.37 µM in the control Scramble cells for the SCC9 lineage.

To further investigate the mechanism by which SIGMAR1 knockdown decreases cisplatin resistance, we evaluated the role of SIGMAR1 silencing in triggering cell apoptosis. Annexin V/PI-positive cells were counted as an index of cell death, at the time of 72 h after treatment of the HN12 cells with 14.57 µM of cisplatin. As expected, the cisplatin treatment induced a significant increase in the apoptosis rate in the shRNA SIGMAR1 + cisplatin (*p* < 0.01) compared to Scramble SIGMAR1 + cisplatin cells (*p* < 0.01), as shown in (Figure 4C). However, it is also observed that SIGMAR1 silencing itself had a direct effect (a modest increase) on apoptosis, which may have caused the increased apoptotic rate in the shRNA-treated cells. Collectively, our findings suggest that SIGMAR1 knockdown enhances the sensitivity of oral cancer cells to cisplatin by promoting apoptosis.

## 3. Discussion

In this study, we aimed to investigate the impact of *SIGMAR1* knockdown in OC cells. *SIGMAR1* is acknowledged as a ubiquitously expressed multifunctional inter-organelle signaling chaperone protein, which plays diverse roles in cellular survival [5]. Notably, any deregulation of *SIGMAR1* expression is associated with tissue instability and can lead to tumorigenesis [6,12]. To our surprise, the role of *SIGMAR1* in oral cancer (OC) biology has not been explored to date. Here, we identified *SIGMAR1* overexpressed in our cohort of human OC samples. The overexpression of *SIGMAR1* has also been demonstrated in breast cancer and colorectal cancer [9,10,13,14]; this is consistent with our results. Notably, the overexpression of this gene was also associated with poor survival rates for patients.

Previously, it was demonstrated that *SIGMAR1* receptors are involved in the immunomodulatory process (immunity and inflammation) and cancer progression [6]. In fact, cancer development is also due to several alterations, including escape from immunosurveillance [15]. In this regard, we hypothesize that *SIGMAR1* may play significant roles in tumor-mediated immune escape, which is a crucial strategy for tumor survival, by inducing *PD-L1* expression. Beyond that, the high expression of *PD-L1* in tumor cells contributes to tumor immune evasion, drug resistance [16], and poor survival rates in oral cancer [17]. Additionally, elucidating these cross-talks between *SIGMAR1* and the tumor cellular microenvironment could significantly accelerate the identification of novel immunological biomarkers for oral cancer treatment.

Firstly, we observed that *SIGMAR1* mRNA expression was positively correlated with the high expression levels of *PD-L1*. Conversely, our results also suggest that *SIGMAR1* is a positive regulator of *PD-L1* expression in human OC because the *SIGMAR1* silencing decreases the abundance of *PD-L1*, at both the total protein and surface expression levels in vitro. Although few studies in the literature demonstrate other mechanisms by which *SIGMAR1* can modulate *PD-L1* expression, *SIGMAR1* may influence *PD-L1* expression indirectly through cross-talk with immune cells in the tumor microenvironment, such as tumor-associated macrophages or T cells, which can secrete cytokines or other factors that regulate *PD-L1* expression in cancer cells. On the other hand, our data were consistent with those of a previous study which revealed that the *SIGMAR1* inhibitor decreased cell surface *PD-L1* expression and suppressed the functional interaction of *PD-1* and *PD-L1* in a coculture of T cells and breast and prostate cancer cells [7].

In this regard, we suggest that the overexpression of *SIGMAR1* is probably a key contributor to chemoresistance and, consequently, tumor cell survival, due the fact that we also observed that the knockdown of *SIGMAR1* decreased chemoresistance to cisplatin and increased apoptosis in human OC cells. Interestingly, our results were consistent with those of an earlier study that also observed that *SIGMAR1* silencing increased the sorafenib sensitivity of hepatocellular carcinoma cells in vitro and in vivo [18]. Notably, our study has two significant limitations: it does not investigate the potential impact of *SIGMAR1* on the sensitivity or resistance to other commonly used oral cancer treatments, and the small number of analyzed samples in our validation study may affect the generalizability of our findings. Future studies should focus on this aspect to better understand the broader implications of *SIGMAR1* expression and functionality in therapeutic contexts beyond the scope of our current research. In this connection, further research is needed to elucidate the specific molecular mechanisms by which *SIGMAR1* modulates *PD-L1* expression and its implications for oral cancer progression and immunotherapy response.

Beyond suggesting potential immunomodulatory roles for *SIGMAR1* in oral cancer, which are reflective of its poorer prognosis, our study provides novel evidence indicating that *SIGMAR1* acts as a positive regulator of *PD-L1* expression, thereby exacerbating chemoresistance in human oral cancer. However, future studies should prioritize elucidating the molecular mechanisms and consequences of *SIGMAR1* silencing on *PD-L1* modulation in vivo. Such efforts are crucial for facilitating the development of potential *SIGMAR1*-targeted interventions, which could be integral for future immunotherapy trials. By transitioning from laboratory discoveries to clinical application, these interventions hold promise as they may directly benefit patients at the bedside.

## 4. Materials and Methods

### 4.1. Sample and Biological Specimens

This study comprised 26 fresh-frozen samples from patients (38–90 years old) diagnosed with OC and their corresponding surgical margins obtained from surgery between 2017 and 2019 at the Instituto do Cancer do Estado de São Paulo (ICESP), Hospital das Clínicas, University of São Paulo Medical School (HCFMUSP), Brazil. This study was approved by the Human Research Ethics Committee of FCFRP-USP and ICESP/HCFMUSP (CEP/FCFRP; CAAE: 90532418.6.0000.5403, ICESP/HCFMUSP CAAE: 90532418.6.3002.0065), and informed consent was obtained from all the patients included. The baseline patient characteristics of the discovery cohort are summarized in Supplementary Table S1. Unfortunately, socio-demographic data were not obtained for the duration of the current study.

### 4.2. Data Source Availability

From the R2: Genomics Analysis and Visualization Platform (http://r2.amc.nl), we downloaded the *SIGMAR1* expression values of the 502 oral cancer tissues and 44 adjacent non-tumor tissues (TCGA data, accessed on 10 January 2024). The raw data used in the study for the analyses of TCGA samples are displayed in Supplementary Table S2.

### 4.3. Cell Lines and Culture Conditions

The OC cell lines (HN12 and SCC9), Human Embryonic Kidney 293T cells (HEK293T), and human oral keratinocytes (derived from non-tumor tissue) spontaneously immortalized (NOK-SI) were used. All the cells were maintained in DMEM/F12 medium (Gibco™, Thermo Fisher^®^, Carlsbad, CA, USA), supplemented with 10% FBS, 100 U/mL penicillin, and 100 μg/mL streptomycin and kept in a humid atmosphere containing 5% CO_2_ at 37 °C.

### 4.4. Lentivirus-Mediated Short Hairpin RNA (shRNA) Knockdown of Gene Expression

Silencing of *SIGMAR1* was performed using the shRNA vector TRCN0000291305, NM_005866. Target Sequence: GACTTCCTCACCCTCTTCTAT, and their respective control Scramble_shRNA—Target Sequence: CCTAAGGTTAAGTCGCCCTCG. Both were acquired from Sigma-Aldrich (St. Louis, MO, USA) and contained a gene for puromycin resistance. Plasmids were expanded in LB medium supplemented with 100 μg/mL of ampicillin and purified using the QIAprep Spin Miniprep Kit protocol (Qiagen Company, Hilden, Germany, #Cat. 27,104), following the manufacturer’s instructions. To analyze the yield and purity of the plasmids, the NanoDrop Spectrophotometer device (Thermo Scientific, Waltham, MA, USA) was used. Lentiviral particles were produced by co-transfection of the trans-lentiviral packaging mix with an shRNA transfer vector into HEK 293T packaging cells (OpenBiosystems, Huntsville, AL, USA). For cell infection, viral supernatants were supplemented with 6 μg/mL polybrene and incubated with the cells for 24 h. Then, the transduced cells were selected with puromycin (0.5 μg/mL) for 5 days.

### 4.5. RNA Extraction, cDNA Synthesis, and Quantitative Real-Time PCR (qRT-PCR)

Total RNA was extracted from an in-house cohort of 26 OC tissues and their corresponding surgical margins, using the AllPrep DNA/RNA/Protein Mini kit (QIAGEN, Hilden, Germany), following the manufacturer’s specifications. RNA concentrations were determined using an ND-1000 spectrophotometer device (NanoDrop 1000 Technologies, Wilmington, DE, USA). The reverse transcription reaction for the synthesis of complementary DNA strands (cDNA) was performed using 100 ng of total RNA and the High-Capacity kit (Applied Biosystems, Foster City, CA, USA) according to the manufacturer’s instructions. Relative mRNA expression levels were measured by quantitative PCR using the GoTaq^®^ qPCR Master Mix (Promega) kit. The reactions were performed on RealPlex4 (Eppendorf), using the internal control: *GAPDH*. The data were analyzed using the 2^−ΔΔCT^ method [19]. The experiments were carried out in triplicate. The primer sequences used in this study, along with their respective thermal cycling conditions, are detailed in Table 1. This table provides an easy reference for all the primers and their specific conditions used during the experiments.

### 4.6. Western Blot

The cells lines were lysed on ice in lysis buffer containing freshly added protease inhibitor cocktail (Roche Diagnostics, Branchburg, NJ, USA). Protein extracts (30 μg) were size-fractionated by SDS-PAGE, and the proteins were immunoblotted with anti-SIGMAR1 (dilution 1:1000, cat. no. #HPA018002, Cell Signaling Technology, Danvers, MA, USA) and anti-PDL1 (dilution 1:1000, cat. no. #13684S, Cell Signaling). Posteriorly, they were normalized with anti-glyceraldehyde 3-phosphate dehydrogenase (GAPDH) or β-actin. All the antibodies were diluted according to the manufacturer’s instructions, and HRP-conjugated goat anti-rabbit (Santa Cruz Biotechnology, Santa Cruz, CA, USA) was used as a secondary antibody. The results were visualized using an enhanced chemiluminescence detection system (Bio-Rad Laboratories, Inc., Hercules, CA, USA), and the relative quantification of the protein level was determined using ImageJ^®^ software (National Institutes of Health, Bethesda, MD, USA). The experiments were carried out in triplicate.

### 4.7. Confocal Microscopy

The cells were seeded on glass coverslips in 24-well plates and fixed with cold methanol for 10 min. In addition, phosphate-buffered saline buffer containing 0.5% (*v*/*v*) Triton X-100 and 3% (*w*/*v*) bovine serum albumin (BSA) was used for blocking for 30 min. The primary antibodies for PD-L1 (#13684S, Cell signaling) were incubated overnight at 4 °C. After incubation with secondary antibody conjugated to AlexaFluor R 488 (dilution 1:1000, #1028736, Invitrogen, Waltham, MA, USA) or DyLight R 650 (dilution 1:1000, #SA-510089, Thermo Scientific, Waltham, MA, USA) for 1 h, the cells were stained with DAPI (D9542, Sigma-Aldrich, St. Louis, MO, USA). A Leica TCS SP8 confocal microscope (Leica Microsystems, Wetzlar, Germany) was used to obtain the images using the 63× objective. Five random fields were captured and ImageJ^®^ software (National Institutes of Health) was used to quantify fluorescence intensity.

### 4.8. Flow Cytometry

A total of 200.000 cells/well were fixed in 4% paraformaldehyde diluted in PBS and permeabilized in PBS containing 1% fetal bovine serum (FBS), 0.2% saponin, and 0.1% sodium azide. The monoclonal antibody PD-L1 (#13684S, Cell signaling) was added, and the cells were incubated for 20 min at 4 °C. After the staining, the cells were washed and fixed in 1% paraformaldehyde diluted in phosphate buffer saline (PBS) and further analyzed in Guava easyCyte 8HT. The data were visualized in t-Distributed Stochastic Neighbor Embedding (t-SNE) density plots generated in FCS express 6 (De Novo software, version 7.18.0015, Pasadena, CA, USA). The experiments were carried out in triplicate.

### 4.9. Cell Viability Assay

Viable cells were quantified using the reagent Alamar Blue (ThermoFisher Scientific, Rockford, IL, USA) according to the manufacturer’s instructions. Briefly, 6 × 10^3^ cells were plated in 96-well plates. The cells were treated with cisplatin at different concentrations (5 µM, 10 µM, 15 µM, 20 µM, and 30 µM) for 72 h, and the results were obtained through the SpectraMax^®^ L Microplate Reader device. Each condition was made in quadruplicate, and the absorbance of 540 and 630 nm was measured using the Epoch 2 Microplate Spectrophotometer (BioTek Instruments Inc., Winooski, VT, USA). The results were determined by the mean of three independent tests. Furthermore, the concentration of cisplatin that inhibited 50% of cell viability (IC50) was determined using the CalcuSyn Software, version 2.0 (Biosoft, Cambridge, UK).

### 4.10. Apoptosis Detection

In total, 17.5 × 10^4^ cells were plated and treated for 72 h with cisplatin at a dose of 14.57 µM to HN12 cells. The detection of cell death was performed by labeling apoptotic cells with Annexin V (APC) (BD Biosciences Pharmingen, San Jose, CA, USA) and propidium iodide (PI). The cells were trypsinized and centrifuged at 1200× *g* for 5 min at 4 °C, washed with ice-cold PBS 1×, and then resuspended in 200 µL of 1× binding buffer (BD Biosciences Pharmingen, San Jose, CA, USA) with 5 µL of annexin-V and 50 µL of a solution of PI (50 μM) and incubated for 15 min, protected from light, at room temperature. The cells were analyzed by BD FACSCalibur TM flow cytometer (BD Biosciences, San Jose, CA, USA). The results were shown as the mean from each condition analyzed in triplicate. The data were analyzed using FlowJo 8.7 software.

### 4.11. Molecular Docking

Molecular dynamics was applied to investigate the potential interaction between SIGMAR1 and PD-L1 in a protein–protein interaction. Thus, molecular docking assays were performed using the ClusPro server (https://cluspro.org), which is a widely used tool for protein–protein docking [20]. The results for the regions of interactions among the proteins and their respective complexed ligands were arranged in an increasing energy order (from most negative to most positive) for the purpose of determining the finest interaction profiles. We also used PyMOL v2.5 as a molecular visualization system.

### 4.12. Statistical Analysis

Graph Prism 5.0 (GraphPad Software, San Diego, CA USA) was used for statistical analyses. The measurement data were presented as mean ± standard deviation (SD). The independent sample *t*-test and the paired sample *t*-test were used. The receiver operating characteristic (ROC) curve was used to assess the prognostic precision of *SIGMAR1* and *PD-L1*. The sensitivity and specificity were measured by the corresponding area under the ROC curve (AUC). The *p*-value < 0.05 indicates statistical significance. The correlations between the *SIGMAR1* and *PD-L1* mRNA levels and *SIGMAR1* with the expression and abundance scores of the immune cells were evaluated by Spearman’s correlation. To better visualize the profile expression of *SIGMAR1* and *PD-L1* in our cohort of human OC samples, we generated a heatmap using the Complex Heatmap package. The *p*-values were considered statistically significant if they were *p* < 0.05.

## 5. Conclusions

Taken together, our results demonstrate that *SIGMAR1* is overexpressed in oral cancer. Additionally, *SIGMAR1* knockdown also reduces *PD-L1* expression and mitigates cisplatin chemoresistance by promoting tumor apoptosis. In this connection, our findings offer, for the first time, experimental evidence suggesting that SIGMAR1 modulators represent a novel class of therapeutic agents with the potential to revolutionize PD-L1/PD-1 blockade strategies in cancer immunotherapy. By targeting PD-L1, these agents offer a complementary approach to monoclonal antibodies, addressing current therapeutic limitations and paving the way for personalized and effective cancer treatments. This novel strategy not only complements existing monoclonal antibody therapies but also offers potential advantages in regulating the tumor immune microenvironment.

## Figures and Tables

**Table 1:** Primer sequences and thermal conditions.

Primer Name	Sequence (5′ → 3′)	Annealing Temperature (°C)
*PD-L1* forward	AAATGGAACCTGGCGAAAGC	63.6
*PD-L1* reverse	GATGAGCCCCTCAGGCATTT	63.7
*SIGMAR1* forward	AGCTCACCACCTACCTCTTTGG	61.9
*SIGMAR1* reverse	ACATGGGCTCCAGCAAGTG	62.3
*GAPDH* forward	GACTTCAACAGCGACACCCACTC	65.5
*GAPDH* reverse	GTCCACCACCCTGTTGCTGTAG	64.1

## Data Availability

The data presented in this study are available in this article (and Supplementary Materials). Additionally, other items that support the results of the study will be made available upon reasonable request.

## Supplementary Materials

The following supporting information can be downloaded at: https://www.mdpi.com/article/10.3390/ijms252211856/s1.

## Abbreviations

Oral cancer (OC), oral squamous cell carcinoma (OSCC), head and neck squamous cell carcinoma (HNSCC), Instituto do Cancer do Estado de São Paulo (ICESP), Hospital das Clínicas, University of São Paulo Medical School (HCFMUSP), Human Embryonic Kidney 293T cell (HEK293T), Western blot (WB), bovine serum albumin (BSA), immunohistochemistry (IHC), Tumor Immune Estimation Resource (TIMER), Kyoto Encyclopedia of Genes and Genomes (KEGG), receiver operating characteristic (ROC), Real-Time Quantitative Reverse Transcription PCR (qRT-PCR), Gene Ontology (GO), tumor-infiltrating immune cells (TIICs).
