# Supplementary material for: SIGMAR1 Knockdown Enhances Oral Cancer Cell Chemosensitivity to Cisplatin via Decreased PD-L1 Expression
Source: Int J Mol Sci. 2024 Nov 5;25(22):11856. doi: 10.3390/ijms252211856 (PMC11594079; doi:10.3390/ijms252211856)
Supplement: Supplementary file 1 [file ijms-25-11856-s001.zip › ijms-3107015-supplementary/ijms-3107015-supplementary.pdf]

Supplementary Table S1. The information of clinicopathological characteristics of patients with OC.

Characteristics	Number of case (n) and (%)
**Age (year)**	
≥59	14 – (54 %)
<59	12 – (46 %)
**Sex**	
Male	20 – (78 %)
Female	6 – (22 %)
**Distant metastasis**	
No	23 - (88 %)
Yes	3 - (12 %)
**Lymph node metastasis**	
No	20 - (77 %)
Yes	6 - (23 %)
**Perineural invasion**	
No	6 - (23 %)
Yes	20 - (77 %)
**Extracapsular invasion**	
No	11 - (43 %)
Yes	6 - (23 %)
NA	9 - (34 %)
**Histology/Desmoplasia**	
Milde	4 - (15 %)
Moderate	14 - (54 %)
Intense	1 - (4 %)
Unknown	7 - (27 %)
**Clinical Stage**	
I-II	3 - (12 %)
III	5 - (19 %)
IV	18 - (69 %)
**Adjuvant Chemotherapy**	
No	20 - (77 %)
Yes	6 – (33 %)
**Adjuvant Radiotherapy**	
No	8 – (31 %)
Yes	18 – (69 %)

**Locoregional Relapse**	
No	25 – (96 %)
Yes	1 – (4 %)
**Disease Status**	
Disease free	19 – (73 %)
Relapse/Metastasis	7 – (27 %)
**Survival Status**	
Alive	17 – (65 %)
Death	9 – (35 %)
**Smoker**	
No	4 – (16 %)
Yes	22 – (84 %)
**Alcoholism**	
No	5 – (19 %)
Yes	21 – (81 %)

NA, Not available.
